# Diagnostic Evaluation of Visual Snow

**DOI:** 10.3389/fneur.2021.743608

**Published:** 2021-09-16

**Authors:** Michael S. Vaphiades, Brendan Grondines, Kasey Cooper, Sean Gratton, Jennifer Doyle

**Affiliations:** ^1^Department of Ophthalmology, University of Alabama, Birmingham, AL, United States; ^2^Department of Ophthalmology, University of Missouri-Kansas City School of Medicine, Kansas City, MO, United States; ^3^Department of Ophthalmology, Little Rock Eye Clinic, Little Rock, AK, United States

**Keywords:** visual snow, visual snow syndrome, MRI, CT, OCT, elecroretinography

## Abstract

**Introduction:** To determine which patients with visual snow (VS) and VS syndrome (VSS) require standard ophthalmologic testing including automated visual field and which patients require further testing such as macular spectral domain optical coherence tomography (SD-OCT), electrophysiology, and neuroimaging.

**Materials and Methods:** We retrospectively reviewed 52 consecutive patients at three institutions with VS and VSS including the University of Alabama, Callahan Eye Hospital, the University of Missouri-Kansas City School of Medicine, and the Little Rock Eye Clinic from the years 2015 to 2021. We collected historical information, examination findings, ophthalmic testing, electrophysiology, and neuroimaging.

**Results:** Of the 52 patients with VS and VSS, eight of the 52 cases met the clinical criteria for VSS. The ages ranged from 7 to 79 years, with a mean age of 25 years (SD = 14.0). There were 22 males and 30 females. Color vision was tested in 51 cases and was normal in 47 cases (92%). A funduscopic exam was performed in all 52 cases and was normal in 46 cases (88%). The macular SD-OCT was normal in all of the 19 cases that it was performed (100%). A Humphrey visual field was performed in 50 cases and was normal in 43 (86%). A visually evoked potential (VEP) was normal in 18 of the 19 cases where it was obtained (95%). The full-field electroretinography (ffERG) was obtained in 28 cases and was normal in 25 (89%). The multifocal electroretinography (mfERG) was normal in 11 of 12 cases (92%). Only four patients accounted for all of the abnormal electrophysiological tests. In the 37 cases that had an MRI, 29 were normal (78%). Only one patient revealed a lesion in the visual pathway (right optic nerve enhancement in an optic neuritis patient).

**Conclusions:** Patients with VS and VSS, if typical in presentation and with normal testing, do not require a workup beyond a thorough history, neuro-ophthalmologic examination, and automated perimetry. If this testing is abnormal, then ancillary testing is required.

## Introduction

Visual snow (VS) is a visual phenomenon that is akin to looking at an old analog television where the reception is poor (1). VS syndrome (VSS) is VS plus other visual and perceptual symptoms (2). VS usually manifests in early life, with black and white, transparent, or different combinations of color static effects. Floaters, afterimages, and photophobia are almost invariably also present (3).

The main question we wanted to answer from this study is if ancillary testing is required in the typical patient who experiences VS symptoms.

Secondarily, since we accumulated more VS and VSS patients than anticipated, we thought it prudent to determine common features in the history, ophthalmologic examination, electrophysiological and ophthalmologic testing, and neuroimaging.

## Methods and Materials

We retrospectively evaluated 52 patients who experienced VS and VSS as defined by in 2014 by Schankin et al. (2) from three institutions including the University of Alabama, Callahan Eye Hospital (MV, BG, and KC), the University of Missouri-Kansas City School of Medicine (SG), and the Little Rock Eye Clinic (JD) from the years 2015 to 2021. Patients underwent a history inquiry (quality and length of the snow) and examination (visual acuity and color testing), ophthalmic imaging (automated visual fields, macular spectral domain optical coherence tomography (SD-OCT), electrophysiological testing consisting of full-field electroretinography (ffERG), multifocal electroretinography (mfERG), and visually evoked potential (VEP)), and brain neuroimaging consisting of magnetic resonance imaging (MRI) and/or computed tomography (CT) of the head. Not every patient underwent every test. Visual acuity was deemed normal if Snellen acuity was 20/25 or better, color vision was deemed normal if all the color plates are identified, and visual fields were deemed normal as reviewed by each physician's interpretation. The head MRI/CT was deemed normal per radiologic interpretation. Medical charts and referral letters were reviewed to identify a previous diagnostic history of psychiatric comorbidities, neurological examination results, and prescribed medications. We also notated race, gender, psychological conditions, migraine headaches, and other comorbidities and if any treatment was instituted. A PubMed literature review using the term “visual snow” and “visual snow syndrome” was performed. References were reviewed and articles discovered.

## Results

Of the 52 patients with VS and VSS, eight of the 52 cases met the clinical criteria for VSS. The ages of the 52 patients ranged from 7 to 79 years, with a mean age of 25 years (SD = 14.0). There were 22 males and 30 females. More than half (27) of all cases were white, four were African American, and there were one each of Hispanic, Asian, and Native Hawaiian descent. In 17 cases, no information on race was reported. Nine patients reported VS for as long as they could remember, 15 reported to have it for the majority of their life, 35 reported that the VS developed later in life, and eight cases reported no information on the duration of symptoms. There were 27 cases with migraine headaches (52%), five of whom reported visual aura, however separate from the VS. Nine patients noted palinopsia, three had nyctalopia, four had floaters, five had photophobia, and seven had enhanced entopic phenomena. Sixteen of the 52 patients reported at least one psychiatric condition (30%). These included 11 cases of depression, eight of anxiety, two of insomnia, one of attention deficit hyperactivity disorder (ADHD), one of bipolar disorder, one of borderline personality disorder, one of post-traumatic stress disorder (PTSD), and one of both Asperger's and Tourette's syndromes.

The best-corrected Snellen visual acuity was 20/25 or 20/20 in 46 of the 52 cases (88%). Color vision was tested in 51 cases and was normal in 47 cases (92%). A funduscopic exam was performed in all 52 cases and was normal in 46 cases (88%). The macular SD-OCT was normal in all of the 19 cases that it was performed (100%).

A Humphrey visual field test was performed in 50 cases and was normal in 43 (86%). Two of the cases with an abnormal Humphrey visual field showed an enlarged blind spot in both eyes (OU), one case was initially normal but when retested 1 year later showed an enlarged blind spot in the right eye (OD) but still normal in the left eye (OS), one case showed central depression OU, one case showed mild constriction OU, and one case was normal OD but showed nasal superior depression OS.

Electrophysiological studies were generally unrevealing. A VEP was normal in 18 of the 19 cases where it was obtained (95%). Similarly, the ffERG was obtained in 28 cases and was normal in 25 (89%). The mfERG was normal in 11 of 12 cases (92%). Only four patients accounted for all of the abnormal electrophysiological tests, and they are as follows: (1) a ffERG showed abnormally depressed OS. This patient had deprivation amblyopia OS secondary to congenital hemangioma of the left upper lid. (2) A mfERG was abnormal. This patient also had pathologic high myopia (−14.00 diopters OD and −13.00 diopters OS), which appeared to be responsible for the abnormal mfERG. (3) A ffERG was abnormally depressed OD. This patient complained of monocular visual loss in that eye. (4) Both the VEP and mfERG showed abnormal OU. This was initially felt to be related to high myopia OU and keratoconus OU; however, the ffERG was abnormal OU as well, which prompted genetic testing revealing a MYO7A variant, which is associated with autosomal recessive Usher type 1. Thus, all four cases had other complaints and exam findings accounting for the abnormalities other than the VS complaint.

Neuroimaging was also generally unrevealing. Overall, 43 patients had cranial neuroimaging: nine had CT, 37 had MRI, and three had both. All nine of the cases that had a CT of the head were normal (100%). In the 37 cases that had an MRI, 29 were normal (78%). The abnormalities among these eight MRI patients ranged from a (1) a right cerebellar hemispheric lesion “scar”, (2) fluid-attenuated inversion recovery (FLAIR) changes in the thalamic and subthalamic regions, (3) tonsillar ectopia and mild ventriculomegaly, (4) areas of periventricular white matter changes, (5) enhancement of the right optic nerve just prior to the chiasm, (6) small amount of fluid in the air cells of the right petrous pyramid, (7) right frontal deep vein abnormality, and (8) left-sided cerebellar venous angioma. Only one patient revealed a lesion in the visual pathway (right optic nerve enhancement in an optic neuritis patient).

## Discussion

VS was first described by Liu et al. in 1995 as an “unusual complication of migraine” manifesting as “persistent diffuse small particles such as TV static, snow, lines of ants, dots, and rain” in the patient's entire visual field (1). It is akin to looking at an old analog television where the reception is poor (2). It generally lasts for months to years, and no underlying etiology is identified. This syndrome was first referred to as “visual snow phenomena” in 2005 (4) and later as VSS (5) with VS as the defining characteristic of the VSS, which includes other visual and perceptual symptoms (2, 6). In 2014, Schankin et al. proposed a definition of VSS to include two of the following: (1) palinopsia, (2) enhanced entopic phenomena (excessive floaters, excessive blue field entopic phenomena, self-light of the eye, or spontaneous photopsia), (3) photophobia, and (4) nyctalopia (night blindness). Also, symptoms cannot be consistent with typical migraine aura, another disorder or medication effect (2). In 2018, these criteria were adopted by the International Headache Society as VSS criteria (7). Patients may experience VS without the complete VSS; and as stated in the above criteria, it is not associated with the effects of psychotropic substances on the brain or other chronic neurological or ophthalmologic disorders (3). Even though VS is not typical of migraine aura, migraine is frequently reported in approximately 70% of patients (3). Migraine headache was present in 52% of our patient population. The perception of VS has been attributed to dysfunctional central sensory processing, which overlaps with, yet is different from, migraine (8).

It is clinically advantageous to have an understanding of what a “typical” patient with VSS experiences. Naturally, the key feature of VSS is the symptom of VS itself: dynamic, continuous, tiny dots in the entire visual field. Typically, the dots are black/gray on a white background or gray/white on a black background; however, the visual phenomena can also be transparent, white flashing, or colored (9), and typically there is no auditory component. Other visual symptoms coexist with VS as part of the syndrome as mentioned to include, but are not limited to palinopsia, enhanced entoptic phenomena, photophobia, and nyctalopia (3). Yoo et al. reviewed the neuro-ophthalmic findings in 20 patients with VSS, and they detected high rates of other visual symptoms including illusionary palinopsia (61%), enhanced entoptic phenomenon (65%), disturbance of night vision (44%), and photophobia (65%) (10). Non-visual symptoms such as tinnitus (7) and even symptoms such as difficulty concentrating and irritability can occur as well (11). The VS typically appears early in life, and in approximately 40% of patients, the symptom has been present for as long as they can remember (3).

Migraine is highly comorbid with VSS (2, 3); however, unlike migraine, VSS does not display a gender prevalence (3) as in our patient population.

These patients typically have a normal neuro-ophthalmologic exam; (10) however, a subpopulation may have atypical history and exam leading to a neuro-ophthalmologic disorder originating from diseases of the eye or the brain. It has been reported in rod-cone dystrophy (10), idiopathic intracranial hypertension (10), Creutzfeldt–Jakob disease (12), and paraneoplastic syndromes (13), among others. It is important for the clinician to distinguish between VS that originates from one of these potentially vision-threatening and dangerous pathologies and idiopathic or “isolated VS or VSS.” A detailed history inquiry is the most effective way of making this distinction. Beyond the history inquiry and exam, it is not well-established whether ancillary testing such as brain imaging or electroretinography is required in the workup of patients presenting with VS. Yoo et al. examined 20 patients with VS, and one was a 36-year-old woman had classic symptoms of VS; however, based on history, the symptoms had only occurred for 6 years, and she had binasal defects on the visual field. This prompted further workup revealing an abnormal ffERG and rod-cone dystrophy (10).

In our population of 47 patients, we obtained a variety of different tests that were all not uniform, partly because there is no well-established guidelines on testing and also the retrospective nature of the study. However, our results indicated that ancillary testing yielded no etiologic pathology when patients presented with “typical” historic and exam features of idiopathic or isolated VS or VSS. We define “typical features” as originating at an early age with the appearance akin to looking at an old analog television where the reception is poor, and a completely normal neuro-ophthalmic examination including normal acuity, pupillary exam, color vision, and automated perimetry. Our “typical” VS and VSS patients were identified based on history, ophthalmologic examination, and ophthalmic ancillary testing, which highlight the importance of these practices; and this suggests that clinicians can accurately identify idiopathic VS and VSS.

A complete understanding of the pathophysiology of VSS is lacking, but it is generally understood to be a disorder of visual processing. Using conventional 1.5-T and 3-T MRI, functional MRI, positron emission tomography, and electrophysiology, several authors have offered explanations including a thalamo-cortical dysrhythmia of the visual pathway (14), hyperexcitation of primary and secondary visual cortices (6), increased saliency of normally ignored subcortical activity (15), or some combination of these mechanisms (16). Advanced neuroimaging and neurophysiological studies have uncovered structural, metabolic, and physiological differences in the brains of patients with VSS. These differences include increased gray matter volume in the left primary and secondary visual cortices, the left visual motion area V5, and the left cerebellar crus (3) and hypermetabolism of the right lingual gyrus (17). Patients with VSS have a higher regional cerebral blood flow than controls over an extensive brain network, suggesting that VSS patients have marked differences in brain processing of visual stimuli, validating its neurobiological basis (18). How these differences fit into the puzzle of VS and VSS pathophysiology is not fully understood; however, collectively, they support the notion that this is a disorder of cerebral visual processing. When understood as such, some of the important features of VS and VSS seem logical. A visual processing disorder would be expected to be present from an early age, to be constant and affecting the entirety of the visual field, and to be generally poorly responsive to conventional pharmacologic therapies. Also, one would expect an association with other visual and perceptual symptoms, but to have normal visual function when measured with standard testing. These are all salient features of VS and VSS and can generally be elicited by careful history inquiry and examination.

Patients with VS and VSS do not generally have abnormalities on examination or ancillary testing (10) as in our patient population. However, although most cases of VS are spontaneous, potential secondary causes should be recognized including post-concussion, post-infection, hallucinogen-persisting perception disorder, idiopathic intracranial hypertension, neoplastic, and posterior cortical atrophy (19). Patients who develop VS after an inciting event or related to an underlying comorbidity may have a better prognosis than those in whom it develops spontaneously (19).

The treatments of VSS were reviewed by Eren et al. on data of 153 patients who were treated with 44 different medications. Only eight of the medications were effective at least once. Of all the medications prescribed, lamotrigine and topiramate had the best results, though they were effective in only 22.2 and 15.4% of patients, respectively (20). Other medications that have been studied include amitriptyline (which may worsen VS), benzodiazepines, acetazolamide, valproate, propranolol, naproxen, and sertraline (19, 20). There is no widely accepted standard treatment for VSS.

Limitations of our study include its retrospective nature. The cases were collected from multiple different providers and centers and therefore not standardized. All of our patients had VS, and eight of the 52 patients met the diagnostic criteria for VSS. We suspect that more of our patients would have met the criteria for VSS if not for the lack of a standardized questionnaire and retrospective nature of the study. The other limiting factor is that our various providers evaluated patients in different ways, and as we learned more about VS, the testing seemed to become more uniform.

To answer the question if non-ophthalmic ancillary testing is required in the typical patient who experiences classic VS and VSS symptoms, it appears as if VS is akin to conditions like acephalgic migraine or even much more remotely like blepharospasm, in that clinicians used to work these patients up until the literature proved no benefit to ancillary studies outside thorough history, examination, and neuro-ophthalmic testing (including pupillary exam, ocular motility, and automated visual field examinations), which should be performed on all VS patients. If etiologies other than typical VS are suspected, one should obtain ancillary testing including OCT, electrophysiology, and cranial neuroimaging.

The key historical features of idiopathic VS and VSS are a non-progressive course and constant snowy visual phenomena that involve the entire visual field OU with onset at an early age. In addition, the presence of other features of VS and VSS including comorbid migraine and photophobia in this setting can help reassure the clinician that there is not a worrisome underlying pathology given a normal thorough neuro-ophthalmic examination including automated perimetry, which is essential in ruling out other eye and brain pathologies.

The diagnostic evaluation of VS and VSS patients should be made on a case-by-case basis; however, we propose that if VS originated at an early age, is non-progressive, and is typical in historical presentation and the patient has a normal neuro-ophthalmologic examination including automated perimetry, then ancillary testing is generally unnecessary.

## Data Availability Statement

The raw data supporting the conclusions of this article will be made available by the authors, without undue reservation.

## Ethics Statement

The studies involving human participants were reviewed and approved by UAB Institutional Review Board for Human Use (IRB). Written informed consent from the participants' legal guardian/next of kin was not required to participate in this study in accordance with the national legislation and the institutional requirements.

## Author Contributions

MV: conceptualization, methodology, data curation, writing—original draft preparation, visualization, investigation, supervision, and writing—reviewing and editing. BG: methodology, data curation, writing—original draft preparation, visualization, investigation, and writing—reviewing and editing. KC: data curation and preparation. SG: data curation, visualization, and investigation. JD: data curation. All authors contributed to the article and approved the submitted version.

## Funding

This work was supported in part by an unrestricted grant from the Research to Prevent Blindness, Inc., New York, NY.

## Conflict of Interest

The authors declare that the research was conducted in the absence of any commercial or financial relationships that could be construed as a potential conflict of interest.

## Publisher's Note

All claims expressed in this article are solely those of the authors and do not necessarily represent those of their affiliated organizations, or those of the publisher, the editors and the reviewers. Any product that may be evaluated in this article, or claim that may be made by its manufacturer, is not guaranteed or endorsed by the publisher.
